# Dietary Intake of Athletes Seeking Nutrition Advice at a Major International Competition

**DOI:** 10.3390/nu8100638

**Published:** 2016-10-14

**Authors:** Sarah J. Burkhart, Fiona E. Pelly

**Affiliations:** School of Health and Sport Sciences, University of the Sunshine Coast, Sippy Downs 4556, Queensland, Australia; fpelly@usc.edu.au

**Keywords:** dietary intake, athlete, international competition

## Abstract

International travel and short-term residence overseas is now a common feature of an elite athlete’s competition schedule, however, food choice away from home may be challenging and potentially impact on performance. Guidelines for dietary intake specific to competition exist for athletes, however, there is little evidence available to ascertain if athletes meet these recommendations during competition periods, particularly when food is provided in-house. During the Delhi 2010 Commonwealth Games, dietitians based in the dining hall recorded 24 h dietary recalls with all athletes who visited the nutrition kiosk. Analysis of dietary intake was conducted with FoodWorks (Xyris Pty Ltd., Brisbane, Australia). Overall, athletes reported consuming a median total daily energy intake of 8674 kJ (range 2384–18,009 kJ), with carbohydrate within the range of 1.0–9.0 g per kg of bodyweight (g/kg) (median = 3.8) and contributing to 50% total energy (TE) (range 14%–79%). Protein and fat intake ranged from 0.3–4.0 g/kg (median = 1.7) to 10–138 g (median = 67 g), and contributed to 21% TE (range 8%–48%) and 24% TE (range 8%–44%), respectively. Athletes reported consuming between 4 and 29 different food items (median = 15) in the previous 24 h period, with predominately discretionary, grains/cereals, meats, poultry, fish, eggs, and meat alternative items. This suggests that dairy, fruit, and vegetable intake may be suboptimal and intake of the micronutrients iron, zinc, calcium, and vitamins A and C may be of concern for a number of athletes.

## 1. Introduction

International travel and short-term residence overseas is now a common feature of an athlete’s competition schedule, however, differing eating arrangements and food options when away from home may influence an athlete’s food choice, and potentially their performance. An athlete needs to consume suitable food and fluid prior to, during, and after competition in order to maximise performance [1,2]. While sport-specific recommendations exist for athletes to ensure that they consume sufficient total energy (TE) to meet requirements, carbohydrate (CHO) to replenish glycogen stores, protein to aid in muscle repair and growth, as well as fluid to stay adequately hydrated [1,3,4,5,6,7,8,9], very little evidence is available to ascertain if athletes meet these recommendations in residence during major international competitions.

While appropriate nutrition is important for performance, investigation into the dietary intake of high performance athletes is limited. Although there appears to be considerable individual variability in dietary intake, the majority of studies to date show that athletes tend on average to meet current evidence-based recommendations for protein, but not CHO [10,11,12,13,14]. This is particularly evident in females [15]. A number of studies have reported that TE intake may also be below expected requirements [14,16,17]. However, these studies are limited by the difficulties experienced when attempting to accurately measure dietary intake. Discrepancies exist between methods of data collection (for example a 24 h recall vs. 7 days weighed food diaries), whether the athlete is in a competition or training phase (or a combination of both), if the athlete is living at home or in a training camp, differing physiological requirements (e.g., strength and power athletes vs. endurance athletes), level of competition, and specific behaviours that may be associated with a particular sport (e.g., methods to make weight in weight-category sports). Assessing dietary intake is further made difficult by the practice of underreporting, where individuals report consuming less food than actual intake [18].

Additionally, the majority of research on dietary intake in athletic populations primarily focuses on quantifying dietary intake in regards to energy and macronutrient content; however, this does not guarantee that the athlete is selecting foods that contribute to a high quality diet. Diet quality is a concept based on the variety and type of foods in an entire diet, the relationship between health status and food groups, and is usually assessed by comparison to national dietary guidelines or similar, and the diversity of choices apparent within the diet [19]. To date, very little research on diet quality within the athletic population exists, with the exception of a comparison of the diets of a select group of Polish athletes to the Swiss Food Pyramid. This study found that athletes did not meet the recommendations for a number of food groups within the food pyramid guide [20]. Sufficient variety of foods from all core food groups is not only important for sports performance; it is often indicative of micronutrient intake and thus linked to prevention of deficiency and decreased risk of chronic disease [21], and therefore warrants investigation.

While literature to date provides some information on dietary intake of athletes during both training and competition phases, limited data is available regarding intake while in residence at international competition events. At major events such as the Olympic (OG) and Commonwealth Games (CG), the majority of athletes and their support team live in a village residence and dine in a large communal dining hall. An extensive range of food is provided free of charge, 24 h a day, for the duration of the competition. While recent data shows that athletes attending these events are generally satisfied with the food provided [22,23], data on actual dietary intake is limited. Only one study has investigated the types of diets (regimens) that are followed by these athletes [24]. Apart from records collected in 1949 [25] and 1964 [26], the most recent and relevant data on dietary intake in this type of environment was collected at the Sydney 2000 OG [23]. This data on apparent consumption within the dining hall suggested that athletes were consuming on average 592 g of carbohydrate (46% TE and on average 7–10 g/kg BM), 202 g (16% TE) of protein and 197 g (35% TE) of fat daily [23], however, no data on individual consumption was collected. Additionally, no data is available on the variety of foods consumed. Therefore, the aim of this research was to describe the self-reported food and dietary intake of athletes who sought professional guidance in regards to their competition diet immediately prior to or during competition at a major international competition.

## 2. Materials and Methods

### 2.1. Data Collection

Australian dietitians (*n* = 4) based at the main dining hall nutrition kiosk at the Delhi 2010 Commonwealth Games recorded consultations with athletes who requested assistance with their competition dietary intake from 23 September to 14 October 2010. Athletes were asked to provide demographic details including gender, sport (and event if appropriate), country representing, country of birth, and highest level of education (no schooling, primary/middle school, senior school, or University or other tertiary institution). Information about past experience at similar events, stage of competition (more than 2 days before event, day before event, day of event, between events, or event completed) and previous nutrition support was also collected. Athletes were asked to report if they had a nutrition competition plan to follow specifically for this event. Dietitians also recorded the purpose of the athletes visit to the kiosk (e.g., weight loss/making weight, weight gain, training or performance nutrition, and clinical issues such as food intolerance/allergy).

The dietitian then collected a recall of quantity and timing of all food, fluids and supplements consumed by the athlete over the previous 24 h period on a standard proforma, and verified intake of all food groups from a provided checklist as per the USDA five-step multiple pass method [27]. General questions about usual intake as per standard diet history were collected. The 24 h recall template was piloted and reviewed by a panel of expert dietitians prior to use at this competition. This was based on a similar template used in other settings [28]. Upon completion of the interview, dietitians were asked to subjectively rate the athlete regarding their expert opinion of the athletes’ dietary intake and nutrition knowledge on a Likert scale of 1 (very poor) to 5 (very good).

### 2.2. Data Analysis

Participants were classified into a sport category (power/sprint, weight category, endurance, racquet, skill, and team) based on the physiological requirements of their sport, and a region/country (western: Including Australia and the British Isles, and non-western: Africa, Caribbean, India and Sri Lanka, and Southeast Asia and the Pacific Islands) based on location and cultural style of eating [22]. Athletes were also grouped based on the reason for requesting advice at the kiosk including: General weight loss and making weight, weight gain, performance/training nutrition, and clinical issues (for example, food allergy/intolerance, gastrointestinal issues).

The 24 h recall data was coded and input into FoodWorks Premium Edition (Version 7, Xyris software, Brisbane, Australia 2013) (FoodWorks) by the primary researcher. Foods consumed within the dining hall were matched to the nutritional analysis for the specific menu items that had previously been coded in FoodWorks. If not consumed from the menu, the item was coded against the most appropriate matching food. As FoodWorks is a database of Australian and New Zealand foods, items were coded into the 2013 Australian Dietary Guidelines (ADG) five core food groups (1) Grains—grain and cereal based foods; (2) Vegetables—vegetables and legumes/beans; (3) Fruit; (4) Dairy—milk, yoghurt, cheese and/or alternatives; (5) Meat and alternatives—lean meats, poultry, fish, eggs, tofu, nuts and seeds) and discretionary foods [21] for the analysis of diet variety (number of choices from each group). Discretionary foods were defined as per the ADG as containing high amounts of saturated fat, added sugars and/or salt, and alcohol (for example, potato chips, biscuits, pizza and fried foods) [21]. Some items were coded as both discretionary and a core food due to the contribution of macro- and micronutrients to the athlete’s diet. For example, a number of discretionary foods were included in the calculation of the main food groups. Cakes/biscuits (*n* = 11), pizza (*n* = 6), Coco-pops™ (*n* = 2), and a muffin (*n* = 1) were included in the calculation of the grains group, as the predominant ingredient is a cereal grain and thus are a source of CHO. All data were cross-checked to ensure consistency and accuracy of coding.

Data were further coded and input into IBM SPSS Statistics (Version 21, IBM Corp., Armonk, NY, USA, 2012) for analysis. Data associations were calculated with the Kruskal–Wallis test, Mann–Whitney *U* test, independent *t* test or ANOVA, depending on normality of data. Statistical significance was considered to be *p* ≤ 0.05 a priori. Results on nutrient intake are presented as median and range of g per kg of bodyweight (g/kg) and as a percentage of total energy intake (% TE). Micronutrient results were compared to the estimated average requirement (EAR), which is the “daily nutrient level estimated to meet the requirements of half the healthy individuals in a particular life stage and gender group” [29] or adequate intake (AI) which is the average daily nutrient intake level based on observed or experimentally-determined approximations or estimates of nutrient intake by a group (or groups) of apparently healthy people that are assumed to be adequate [29]. Each athlete’s 24 h recall data was also compared to recommendations [3,4,5,6,7,9,30] for CHO, protein, and fat intake, specific to type of sport and demographic information (for example, height, weight, age, and gender). Diet variety and dietitians’ rating of dietary intake and nutrition knowledge is presented as a median score and range.

### 2.3. Ethical Approval

Ethical approval was granted by the University of the Sunshine Coast Human Ethics Committee (A/10/253). Participation was voluntary and participants were considered to have given consent to participate by taking part in a consultation.

## 3. Results

### 3.1. Participant Characteristics

A total of 44 athletes completed a 24 h dietary recall at the nutrition kiosk, representing 1% of the total number of athletes who competed at this event (*n* = 4352). However, not all athletes reside in the village, eat within the dining hall, and thus have access to the nutrition kiosk. This cohort was the entire sample of athletes that sought dietary advice. Athletes were predominately from non-western regions and reported competing in 13 specific sports (Table 1). Over half of the athletes reported being in a precompetition stage (*n* = 30, 68%), with the majority of these athletes greater than 2 days away from competition (*n* = 28, 82%). The mean self-reported body weight of the male and female athletes was 74 kg (range 56–113 kg) and 65 kg (range 49–101 kg), respectively. Six athletes (14%) reported that they had received nutrition education prior to attending this event. While four athletes (10%) reported having a competition plan to follow, only two of these reported nutrition education prior to this event.

### 3.2. Dietary Intake and Eating Behaviours

Overall, athletes reported consuming a median total daily energy intake of 8674 kJ (range 2384–18,009 kJ), with CHO within the range of 1.0–9.0 g/kg (median = 3.8 g/kg) and contributing to 50% TE (range 14%–79%). Protein intake ranged from 0.3 to 4.0 g/kg (median = 1.7 g/kg) and contributed to 21% TE (range 8%–48%), while total fat intake ranged from 10 to 138 g (median = 67 g) and contributed to 24% TE (range 8%–44%). Dietary intake varied according to reason for requesting assistance at the kiosk and gender of athletes (Table 2). Those competing in racquet (*n* = 6) and power/sprint sports (*n* = 9) reported consuming the greatest energy (median = 11,298 kJ, range 7032–13,485 kJ and 10,149 kJ, range 2472–18,009 kJ, respectively). Athletes competing in skill and power/sprint sports reported consuming the highest protein intake (median = 1.8 g/kg, range 1–3 g/kg, and median = 1.7 g/kg, range 0.5–4 g/kg, respectively), while athletes in team sports (*n* = 2) reported the lowest energy intake (median = 7274 kJ, range 5953–8596 kJ). Athletes in team (*n* = 2) and weight category sports (*n* = 13) reported the lowest contribution of energy from CHO (median = 2.5 g/kg, range 1.7–3.3 g/kg, 39% TE, and median = 3.0 g/kg, range 1.0–6.0 g/kg, 46% TE), while athletes competing in endurance sports (*n* = 4) reported consuming the lowest amount of fat (median = 51 g, range 35–72 g, 21% TE). There was no significant difference in nutrient intake between those in the pre-versus postcompetition phases.

Three main meals were consumed by the majority of athletes (*n* = 23, 77%). Overall, the greatest median contribution to total energy intake was from breakfast (29%) to lunch (31%). Carbohydrate contributed a greater proportion to total energy at breakfast (56%) and to snacks (69%) than lunch and dinner. Protein contribution was predominately from meals consumed at lunch and dinner. Fourteen athletes reported consuming sports drinks as a snack, with these drinks contributing to over half of the TE consumed between meals. Five athletes reported not consuming any snacks in the precompetition period. The average contribution of macronutrients to the total daily intake was similar between genders (Table 3).

**Figure 1:**
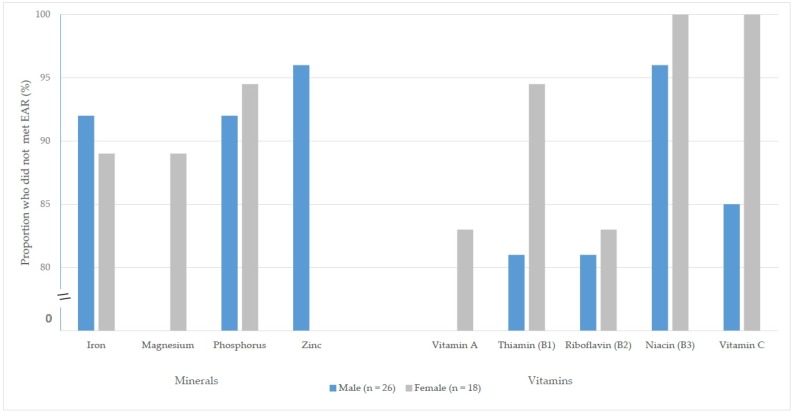
Proportion of male and female athletes who did not meet the micronutrient Estimated Average Requirement (EAR) ^#^. ^#^ Micronutrients displayed are those where greater than 80% of the cohort were below the EAR. The following EAR for 19–50 years old were used: iron male = 6 mg/day , iron female = 8 mg/day; magnesium male 330 mg/day (19–30 years of age), 350 mg/day (31–50 years of age), female 255 mg/day (19–30 years of age), 265 mg/day (31–50 years of age); phosphorus male = 580 mg/day, female = 580 mg/day; zinc male = 12.5 mg/day, female = 6.5 mg/day; vitamin A male = 625 µg/day, female = 500 µg/day; thiamine (B1) male = 1.0 mg/day, female = 0.9 mg/day; riboflavin (B2) male = 1.1 mg/day, female = 0.9 mg/day; niacin (B3) male = 12 mg/day, female = 11 mg/day; vitamin C male = 30 mg/day, female = 30 mg/day. 2 athletes missing data to calculate EAR.

Dietary analysis showed that 80% of all athletes did not meet the estimated average requirement (EAR) for at least one micronutrient in the previous 24 h, with 25% not meeting the EAR/AI for 5 or more nutrients. Greater than 80% of both genders did not meet the EAR for iron and phosphorus, and vitamin B1–B3 and vitamin C. A greater proportion of women and men did not meet the EAR for vitamin A and magnesium, and zinc respectively (Figure 1). Over two-thirds (*n* = 39, 90.5%) did not meet the EAR for iron (female M = 13.66 mg, range 3.8–28; male M = 12.32 mg, range 4.2–25 mg). Overall, *n* = 22 (50%) reported using at least one type of supplement, with almost half (*n* = 19, 43%) of all athletes reporting the use of a vitamin or multivitamin supplement. Multivitamin supplements were not disclosed in the 24 h recall by any athletes, and therefore were not included in the calculation of dietary intake. Five athletes (11%) reported that they had been previously diagnosed with a nutrient deficiency (one from each of team, skill, power/sprint, weight, and endurance), of which 4 of these reported as iron deficiency anaemia (*n* = 1, unknown).

### 3.3. Food Variety

#### 3.3.1. Number of Total Food Items Consumed

Overall, athletes reported consuming between 4 and 29 different food items (median = 15, range 4–29) in the previous 24 h period, with a broad range of items chosen from the discretionary (median = 4, range 0–10), grain (median = 3.5, range 0–7), and meat (median = 3, range 0–7) groups. Athletes from racquet (median = 20, range 15–24) and team sports (median = 18, range 16–20) reported consuming a greater number of food items than other sports in the previous 24 h (Kruskal–Wallis test, *p* = 0.034).

#### 3.3.2. Number and Variety of Items from Each Food Group

Females reported consuming a greater number (median 3.5 vs. 2.0) and variety (3 vs. 1.5) of fruit choices than males (*p* = 0.005 and *p* = 0.001, respectively). Athletes requesting advice for weight gain reported consuming significantly less variety of fruit items than athletes requesting advice for clinical issues (*p* = 0.038) (Table 4). In addition, athletes competing in weight category sports reported consuming less items from the grains group (median 1.0, range 0–5, *p* = 0.028), as well as a lower variety of grains (median 1.0, range 0–3, Kruskal–Wallis test, *p* = 0.017) compared to other sports. There was no significant difference between athletes from western and non–western regions.

#### 3.3.3. Dietitians Rating of Dietary Intake and Nutrition Knowledge

Overall, dietitians rated the dietary intake and nutrition knowledge of athletes as “average” (median 3, range “very poor–good”) and “poor” (median 2, range “very poor–good”), respectively (Table 4). There were no significant differences in international regions or gender. Differences in rating were observed depending on reason for requesting assistance (Table 4). Athletes who requested assistance for weight gain and making weight/weight loss (both median 3 “average”, range “very poor–good”) were rated as having a significantly poorer dietary intake than those requesting performance nutrition (median 3.5 “average–good”, range “poor–good”) and clinical advice (median 3 “average”, range “very poor–good”) (Kruskal–Wallis test, *p* = 0.03).

## 4. Discussion

This research provides a unique insight into the food selection and dietary intake of athletes at a major competition. Although this is based on the results of a small selection of athletes, this is the entire sample of those who actively sought expert advice from dietitians at this event. Our results indicate that there is considerable variability in the dietary intake of these athletes, and despite representing a single day of intake, many report consuming inadequate total energy and CHO for both basic health requirements and athletic performance. The large variation in intake seen in our results may have to do with differing cultural background, sport category, gender, stage of competition and previous professional advice on dietary intake. In addition, our results indicate that many athletes did not consume a varied diet, with some athletes consuming as little as four or five different items in the previous 24 h period, with many not eating items from a range of food groups. While an athlete can appear to meet macronutrient recommendations based on quantifying intake, the variety of food that is consumed may not provide adequate intake of the micronutrients, fibre, and other food components. Inadequate dietary intake was conferred by the dietitians’ general subjective perception of the nutrition knowledge of athletes as poor and dietary intake as average. 

In general, we found that the CHO intake of athletes in this study was similar to that observed previously in similar samples [10,11,31], and supports other literature reporting that athletes from varying sports may not consume enough CHO to meet current recommendations for performance [10,11,12,13,14]. While our results are based on one 24 h recall, and we recognise that this may not be indicative of actual habitual intake, there are a number of potential implications on an athlete’s performance, as inadequate CHO may compromise storage of muscle and liver glycogen, and may in turn affect physical and/or mental performance. Athletes in this study appeared to consume an adequate amount of protein based on a g/kg BW measure, however as we did not investigate the quantity and timing of protein intake in relation to competition, and this would be worth further investigation. The average contribution of fat to dietary intake varied considerably, but can be considered acceptable for general health [1]. It is also similar to that seen in other athletic populations [10,15,31,32,33]. The high consumption of discretionary food items appears to have contributed to fat intake, with foods such as pizza, ice cream, biscuits, cake, and muffin commonly consumed by these athletes. The main gender differences appeared to be around the consumption of a variety of fruit, which is not surprising as it is recognised that males typically consume less servings of fruits and vegetables per day than females [34].

We found those athletes that were attempting to make or lose weight were the predominant group to seek advice on their dietary intake. These athletes reported the lowest TE intake of all sports groups and, while not significantly different, appeared to consume less variety of foods with some individuals reporting no foods consumed from a range of food groups. These athletes are required to weigh in at a certain weight in order to participate in their event, and have been reported to reduce their CHO, protein, and fluid intake before competition in a similar environment [23]. A number of these athletes specifically requested assistance for making weight, suggesting that they were under pressure to lose weight within a short time period, and may therefore have already limited their consumption of food. Athletes, particularly those attempting to make weight, may also have inadequate intramuscular stores of CHO after restricting intake, and an insufficient time period in which to try and restore these before competition for maximal performance. It is possible that this may be linked to a lack of professional guidance on dietary intake, as we found that only 5 of the 26 athletes requesting assistance for making weight had reported having dietary advice prior to this event and a nutrition plan to follow. Furthermore, nutrition knowledge of these athletes was rated as poor by the dietitians.

Given the varied intake of TE and macronutrients, and the poor variety of foods consumed in our sample, it is not surprising that a large number of athletes did not meet the EAR/AI for various micronutrients based on their intake in the previous 24 h period. Previous research suggests that athletes generally tend to consume sufficient food to have an adequate intake of most micronutrients with some exceptions in specific athletic groups for vitamin E [11,33], zinc [14], vitamin A, and iron [17]. Interestingly, we found that iron, phosphorus, vitamin C, and the B vitamins were below the EAR in most athletes in this sample. It is likely that our sample had a poorer intake than the rest of the athlete population, as suggested by their reason for seeking a consultation and the dietitians’ subjective assessment of their intake. No athlete reported consuming supplements in the previous 24 h, however, half of the athletes reported generally consuming a multivitamin (MV) supplement as part of their normal routine. Clearly, some athletes had poor dietary intake and nutrition knowledge, and a lack of variety in their diet. This may place the athletes at increased risk of illness, particularly when combined with the stress of travel and competition in a foreign country.

While we did not detect any differences in athletes’ diet pre- and postcompetition, the phase of competition may actually change eating behaviours. It is plausible that an athlete will be more focused on meeting nutritional goals prior to, as compared to after, competition [35]. Anecdotally, athletes have been known to “relax” their attitude to eating for performance once their event is over, and have been seen to indulge, or consume foods that they may have avoided prior to competition. While this was not apparent in our sample, this may be due to the characteristics of the athletes who participated in this study.

The unique environment of the athlete’s village and the location of training/competition venues at this event may have also influenced our results. The majority of athletes must leave the village and travel to different locations to train or compete. Anecdotally, we noted that a number of comments were made about unsuitable food and snack items being available at various training and competition venues. This may have also led athletes to consume more food before travelling away from the village. There may also be differences in the TE consumed at the dinner meal, as a number of competition events were held at night and this may have influenced when, or if, an athlete could eat this meal.

Additionally, while we asked athletes about stage of competition, we did not ask how long the athlete had been based in the athlete’s village. If the athlete had recently arrived in the athlete’s village, they may have been experiencing jet lag or a loss of appetite on arrival [36]. Conversely, an athlete who has been based in the athlete’s village for a longer period of time may be experiencing menu boredom [22] which may influence food choice. Athletes may also vary from their usual intake as the novelty of attending an elite competition event and living in an athlete’s village may distract them from focusing on nutritional goals [36]. A large proportion of athletes (79%) reported that this was their first experience at this type of event. It is possible that athletes with previous experience in this environment may find it easier to locate appropriate items and deal with the challenges of eating in a communal setting. Another important characteristic of the dining hall is the influence of other individuals on the athletes food choice [35,37,38]. Research shows individuals who are dining with strangers will consume less food than usual, while those who dine with familiar individuals tend to consume more [38]. Further research on the influences on food choice in this environment would provide valuable information to those who work with athletes competing at these events.

It is important to note that this sample of athletes were recruited when they approached the nutrition kiosk for assistance, thus may not have known how to choose appropriate foods for their particular sport. A large proportion of the athletes who took part in this study were from less westernised countries. Previous research at this [39,40], and similar events [23,41] has demonstrated that athletes from these regions are more likely to seek nutritional support in this environment. We noted that only six athletes had received professional nutrition advice prior to this event, so a lack of sports-specific nutrition knowledge may also be a reason for the variability in dietary intake. Interestingly, the dietitians did not rate the dietary intake of athletes from western and non-western regions differently.

There are a number of limitations in this research that need to be acknowledged. Our results are specifically focused on athletes who approached the kiosk for a consultation and therefore do not represent every athlete present at this event. While the dietitians used a predesigned form to conduct the 24 h recall, there may have been differences in recording methods, and the subjective rating given for both the assessment of dietary intake and nutrition knowledge. As with any collection of dietary intake data, there are limitations with the use of the 24 h recall method. While this method is quick and can provide in-depth information about dietary intake [42], it is only a measure of a single day, and does not represent usual intake [18,43]. There is also the potential for underreporting, as some individuals may report consuming less than their actual intake [18]. It is also feasible that the menu items did not reflect the original recipe, resulting in inaccurate nutritional analysis. Due to the nature of the data that was collected, we were not able to score diet quality nor determine exact servings of each food group, but were able to provide an indication of variety of items consumed in the diet, and variety of items from within each food group.

### Future Directions

Based on the results of this study, further research on the dietary intake of athletes in this type of environment is warranted. It would be of interest to investigate what athletes consume over a longer period (both before and during competition) with more detailed methods of data collection. Further research with a larger sample would be beneficial, particularly regarding dietary intake and eating behaviours (e.g., snacking, use of sports drinks, diet quality). As food choice is complex, further research on the factors which influence food choice in this environment would provide insight for caterers and dietitians working with individual athlete.

## 5. Conclusions

Athletes who requested assistance at the nutrition kiosk at a major international competition generally had a poor variety of foods, distribution of, and in some cases inadequate intake of, energy, macro-, and micronutrients. Of particular concern was the dietary intake of athletes who were attempting to make weight or lose weight in the days prior to competition. While this data is limited in that it is only a measure of one day’s intake and is based on the athletes recall, this highlights that these athletes may not be consuming a diet that will assist with maximising performance, and if the same dietary habits are followed over a prolonged period of time, health may also be compromised. Further research is required to examine the dietary habits of athletes in this unique environment.

## Figures and Tables

**Table 1:** Demographic information of all athletes who took part in a 24 h recall.

Demographic	TOTAL *n* = 44	Gender		Reason for Consultation ^@^			
		Female *n* = 18 (41%)	Male *n* = 26 (59%)	Making Weight or Weight Loss *n* = 26 (59%)	Weight Gain *n* = 4 (9%)	Performance *n* = 9 (21%)	Clinical ^#^ *n* = 5 (11%)
**Age (years) (M ± SD)**	26.6 (8)	27.6 (10)	25.9 (7)	25.9 (7)	24 (9)	30.9 (9)	24 (9)
Region * (*n*, %)							
Non-Western ^^^	36 (86)	14 (78)	24 (92)	26	4	9	2
Western ^$^	8 (14)	4 (22)	2 (8)	-	-	-	3
**Sport (*n*, %) ^~^**							
Endurance	4 (9)	2 (11)	2 (8)	2	-	1	1
Power/Sprint	9 (21)	7 (39)	2 (8)	6	-	1	2
Racquet	6 (14)	2 (11)	4 (15)	1	1	3	1
Skill	10 (23)	4 (22)	6 (23)	6	-	3	1
Team	2 (5)	2 (11)	-	1	-	1	-
Weight	13 (30)	1 (6)	12 (46)	10	3	-	-
**Education (*n*, %)**							
Middle/Senior School	7 (17)	2 (12)	5 (20)	4	1	-	2
Completed Senior School	17 (40)	6 (35)	11 (44)	12	1	4	-
Attended University	18 (43)	9 (53)	9 (36)	9	1	5	3
**Experience (*n*, %)**							
First CG/OG	34 (79)	15 (83)	19 (73)	21	3	5	5
First athletes village	30 (70)	14 (78)	16 (62)	18	2	5	5
**Previous nutrition assistance (*n*, %)**							
	6 (14)	2 (12)	4 (16)	5 (20)	0	0	1 (20)

* 2 responses unknown for region and level of education; ^#^ Clinical consultations included Coeliac disease, corn allergy, nut allergy and reflux; ^^^ Bahamas, Bangladesh, Belize, Gambia, India, Kenya, Malawi, Sierra Leone, Sri Lanka, St Vincent, Tanzania, Tonga, Trinidad and Tobago; ^$^ Australia, England, Falkland Islands, Guernsey; ^@^ Proportions not calculated for region, sport, education or assistance as numbers in most categories are <10; ^~^ Endurance includes; athletic events 800 m and over, cycling and swimming distance events; Power/Sprint includes; athletic events under 400 m, athletic field events and swimming sprint events; Racket includes; badminton, table tennis and squash; Skill includes; archery and shooting; Team includes hockey; Weight includes; boxing, weight lifting and wrestling.

**Table 2:** Energy and macronutrient intake of athletes.

Energy and Macronutrients	TOTAL (All Athletes) Median, Range	Gender Median, Range		Reason for Consultation Median, Range			
		Female (*n* = 18)	Male (*n* = 26)	Making Weight or Weight Loss (*n* = 26)	Weight Gain (*n* = 4)	Performance (*n* = 9)	Clinical ^#^ (*n* = 5)
**Energy**							
kilojoules/day	8674, 2384–18,009	8484, 2473–18,008	9369, 2384–15,175	8632, 2384–18,009	11542, 9622–14,560 *	10798, 5953–14,908	7795, 2473–8091 *
**Carbohydrate**							
g/day	241, 68–576	244, 81–576	230, 68–512	239, 68–576	342, 208–512	317, 133–478	198, 81–258
g/kg **	3.8, 1.0–9.0	4.2, 1–9	3.5, 0.1–7	3.6, 0.1–9.0	4.3, 4.3–6.2	4.8, 1.7–7.4	3, 1.3–4.6
% TE	50, 14–79	49, 35–64	51, 14–79	50, 14–79	49, 36–57	52, 36–62	49, 38–53
**Protein**							
g/day	121, 20–276	109, 30–276	127, 20–231	115, 20–276	164, 129–232	120, 55–169	109, 30–163
g/kg **	1.7, 0.3–4	1.7, 0.5–4	1.6, 0.3–3.0	1.6, 0.3–4	1.8, 1.7–2.1	1.6, 0.8–2.1	1.7, 0.5–1.8
% TE	21, 8–48	20, 8–40	22, 9–48	21, 8–48	23, 17–41	18, 10–38	24, 19–40
**Fat**							
g/day	67, 10–138	65, 13–129	67, 10–138	70, 10–138	79, 57–91	74, 28–115	36, 13–56
% TE	24, 8–44	25, 17–43	22, 8–44	25, 8–44	22, 21–31	25, 16–39	20, 17–27

* Energy, kilojoules/day and reason for consultation (Kruskal–Wallis test, *p* = 0.047); ** *n* = 41 as weight unknown for three athletes; ^#^ Clinical consultations included coeliac disease, corn allergy, nut allergy, and reflux.

**Table 3:** Distribution of energy and macronutrient contribution at meal periods.

Distribution of Energy at Meal Time	TOTAL (All Athletes) Median, Range	Gender Median, Range		Reason for Consultation Median, Range			
		Female (*n* = 18)	Male (*n* = 26)	Making Weight or Weight Loss (*n* = 26)	Weight Gain (*n* = 4)	Performance (*n* = 9 )	Clinical ^#^ (*n* = 5)
**Breakfast**							
% TE	29, 8–84	26, 8–55	33, 8–84	33, 8–84	34, 16–35	27, 18–40	19, 15–38
% E CHO	56, 22–87	63, 36–87	54, 22–82	55, 22–87	54, 41–59	55, 28–70	71, 63–83
% E PRO	16, 5–39	15, 6–37	16, 5–39	14, 5–39	18, 12–24	17, 13–23	13, 6–18
% E FAT	23, 2–54	22, 3–46	25, 2–54	23, 2–54	32, 12–35	27, 9–50	11, 3–13
**Lunch**							
% TE	31, 0–72	32, 2–45	30, 0–72	30, 0–72	28, 4–33	32, 18–51	36, 11–45
% E CHO	48, 4–94	45,–81	51, 5–94	46, 5–81	52, 17–94	48, 14–74	35, 4–62
% E PR	26, 2–66	22, 4–55	26, 2–66	28, 4–58	29, 2–66	21, 8–51	34, 7–55
% E FAT	22, 2–48	26, 5–48	17, 2–46	23, 5–45	15, 12–35	17, 11–46	25, 7–48
**Dinner**							
% TE	26, 0–49	28, 0–49	24, 0–44	25, 0–48	22, 0–42	28, 0–40	28, 0–49
% E CHO	46, 12–85	43, 15–76	49, 12–85	45, 12–85	23, 21–70	50, 15–61	33, 26–76
% E PR	22, 5–62	25, 8–55	22, 5–62	22, 5–49	58, 17–62	19, 15–55	30, 8–44
% E FAT	25, 2–53	28, 5–53	18, 2–50	26, 2–53	16, 7–17	27, 10–43	25, 5–38
**Snacks**							
% TE	9, 0–54	10, 0–39	9, 0–54	9, 0–39	21, 9–54	7, 0–50	17, 0–31
% E CHO	69, 35–100	72, 35–100	66, 35–100	69, 35–100	54, 41–80	69, 36–100	71, 57–73
% E PR	8, 0–30	6, 0–20	8, 0–30	6, 0–30	10, 5–15	7, 0–13	9, 6–14
% E FAT	18, 0–56	18, 0–53	18, 0–56	18, 0–56	32, 9–41	18, 0–50	14, 8–30

^#^ Clinical consultations included coeliac disease, corn allergy, nut allergy and reflux.

**Table 4:** Variety of items consumed from each food group and dietitians rating of diet and nutrition knowledge based on gender and reason for requesting assistance.

Variety of Items from Each Food Group ^&^	TOTAL Median (Range)	Gender Median (Range)		Reason for Consultation Median (Range)			
		Female (*n* = 18)	Male (*n* = 26)	Making Weight or Weight Loss (*n* = 26)	Weight Gain (*n* = 4)	Performance (*n* = 9)	Clinical ^#^ (*n* = 5)
Grains	2 (0–6)	2 (0–5)	2 (0–6)	2 (0–5)	2.5 (2–5)	3 (1–6)	2 (0–3)
Vegetables	2 (0–9)	2 (0–9)	2 (0–8)	2 (0–9)	4 (2–8)	4 (0–5)	2 (0–8)
Fruit *	3 (0–8)	3 (0–8)	1.5 (0–4)	2 (0–7)	1.5 (0–2)	2 (0–8)	4 (3–7)
Dairy	1 (0–2)	1 (0–2)	1 (0–2)	1 (0–2)	1 (1–2)	1 (0–2)	1 (0–2)
Meats	2 (0–7)	2 (0–6)	2 (0–7)	2 (0–7)	2.5 (2–4)	3 (1–6)	2 (1–2)
Discretionary ^^^	3 (0–7)	3 (1–7)	3 (0–6)	3 (0–7)	4.5 (3–6)	2 (0–6)	3 (1–3)
TOTAL	15 (4–49)	16.5 (7–29)	13.5 (4–24)	13 (4–21)	15 .5 (14–24)	16 (8–29)	15 (9–22)

^#^ Clinical consultations included coeliac disease, corn allergy, nut allergy, and reflux; ^^^ Discretionary foods included: cakes/biscuits/muffin/pastries, pizza, coco-pops™, soft drinks, ice cream, desserts, and chocolate spread; * Significant difference in fruit variety between genders (Mann–Whitney U test *p* = 0.001) and reason for consultation (Kruskal–Wallis test, *p* = 0.038).
